# Bouveret Syndrome: A Systematic Review of Clinical Factors Associated With Surgical Management

**DOI:** 10.7759/cureus.108109

**Published:** 2026-05-01

**Authors:** Maximus S Reese, Salma Alkhatib, Philip J Haynos, Garret Rymer, Noor Chughtai, Jared Nichols

**Affiliations:** 1 Medical School, Kansas City University, Joplin, USA; 2 Osteopathic Manipulative Medicine, Kansas City University, Joplin, USA

**Keywords:** complications of gallstone disease, gallstone development, gallstone diseases, gallstone extraction, gallstone formation, gallstone impaction, gallstone size, gallstone treatment, impacted gallstone, minimally invasive gallstone surgery

## Abstract

Bouveret syndrome is a rare form of gallstone ileus characterized by the formation of a cholecystoenteric fistula and subsequent gallstone migration into the gastrointestinal tract, resulting in gastric outlet obstruction. Diagnostic criteria consist of clinical evidence (persistent non-bilious vomiting, nausea, epigastric pain) paired with imaging (CT abdomen showing ectopic gallstone, gastric outlet obstruction, cholecystoenteric fistula) and endoscopy. Current expert consensus recommends initial endoscopic stone removal as first-line therapy to avoid techniques associated with higher morbidity and mortality. This review focuses on identifying recurrent findings in clinical and stone-related factors observed in endoscopic failure to determine any features that may guide initial therapy selection. This systematic review analyzed 30 published case reports with 31 patients, consisting of only patient-level clinical data from studies in English. Data was extracted via full-text review, organized into comparative tables, and analyzed for common patterns in symptoms, diagnostic techniques, and stone size in failed endoscopic and successful endoscopic cases. Patients ranged in age from 31 to 92 years and were predominantly female. Therapeutic endoscopic management was attempted initially in 21 cases, with successful extraction in 7 cases. The remaining 14 cases required conversion to open surgical intervention. The mean stone size did not differ substantially between successful and failed endoscopic cases. In this review, stone size did not successfully predict endoscopic success. Bouveret syndrome is a challenging condition to both diagnose and manage, with a high rate of endoscopic failure. Minimally invasive approaches in endoscopy and lithotripsy are appropriate initial strategies in selected patients; however, a substantial proportion ultimately require more open surgical intervention. Consideration of open surgical management may be warranted in patients with more severe obstruction or advanced disease, highlighting the importance of individualized treatment and standardized reporting.

## Introduction and background

Introduction

Bouveret syndrome is a rare variant of gallstone ileus representing 1-3% of gallstone ileus cases and 0.3-0.5% of cholelithiasis complications, making it an uncommon cause of gastric outlet obstruction [1,2]. It occurs when a gallstone migrates from the gallbladder into the gastrointestinal tract through a biliary-enteric fistula. Fistula formation can occur between multiple structures - more commonly, a duodenal (cholecystoduodenal) fistula appears or, less frequently, a biliary-gastric (cholecystogastric) fistula. These fistulas arise due to chronic or recurrent cholecystitis, in which sustained inflammation and pressure from a gallstone against the gallbladder wall lead to ischemia, necrosis, and eventual erosion of the stone through the wall [3,4]. Concurrent inflammation of the gallbladder promotes adhesion to adjacent gastrointestinal structures, allowing stone migration into the enteric tract [4,5].

In typical gallstone ileus, the stone travels distally into the small intestine to cause obstruction at the ileocecal junction. In contrast, Bouveret syndrome involves a more proximal fistula formation resulting in gastric outlet obstruction from stone impaction in the duodenum or pylorus [6-8]. Similar to a conventional gallstone ileus, Bouveret syndrome may demonstrate Rigler’s triad - pneumobilia, ectopic gallstone, and intestinal obstruction; however, Bouveret syndrome differs in its clinical presentation, as it may manifest with features of gastric outlet obstruction, including hematemesis, melena, and bilious vomiting rather than the symptoms typical of distal small bowel obstruction [8,9].

Limited available evidence makes optimal management challenging, particularly given the diagnostic difficulty, advanced age of patients, and significant comorbidities. Endoscopic intervention is frequently attempted as an initial approach due to its minimally invasive nature; however, failure rates >50% often necessitate invasive operative intervention [10]. When endoscopic therapy fails or is not feasible (due to patient risk, anatomical obstruction), surgical intervention may include laparotomy with enterotomy or gastrotomy, with possible addition of adjunctive procedures such as cholecystectomy, fistula repair, or omentoplasty [10-12]. This review provides a descriptive synthesis of reported cases of Bouveret syndrome and characterizes the clinical presentations, imaging findings, and treatment modalities observed. Emphasis is placed on trends in cases of successful endoscopic stone removal and in cases of failure, with the goal of describing patterns and generating hypotheses rather than establishing factors that influence management decisions or determine the need for surgical intervention. 

Methods

A literature search of published case reports on Bouveret syndrome was performed, focusing on studies involving patients with confirmed cholecystoduodenal or cholecystogastric fistulas. The search was conducted using PubMed from database inception to March 27, 2026, and Embase from database inception to March 27, 2026, with the final search performed on March 27, 2026. The following Boolean search strategy was applied: (“Bouveret syndrome”[Title/Abstract] OR “Bouveret's syndrome”[Title/Abstract]). The search term was selected to capture explicitly labeled cases of Bouveret syndrome. Results were filtered to include only published reports within the past 10 years and limited to human subjects. A 10-year restriction was applied to reflect contemporary techniques. No language restrictions were initially applied.

This search retrieved a total of 130 articles in PubMed and 130 articles in Embase. Embase results were screened following completion of PubMed screening to identify additional eligible studies. Duplicate articles between the databases were identified and removed. Three reviewers independently screened individual titles and abstracts to determine if articles were eligible to be advanced to full-text review. Any potential discrepancies were to be resolved via consensus discussion; however, no disagreements occurred. A reference list of included articles was manually reviewed. Inclusion criteria consisted of case reports and case series describing confirmed cases of Bouveret syndrome with accurate recording of patient-level data, including demographic information, presenting symptoms, diagnostic techniques utilized, clinical and surgical management, gallstone size, and outcome data. After full-text assessment, 30 studies comprising 31 patients were ultimately selected. Additional studies of Bouveret syndrome were identified but excluded due to a lack of detailed clinical presentation, management strategy, or patient outcome reporting. Non-English reports without translation were excluded. Variability in treatment approaches was retained for comparative analysis between gallstone sizes, surgical techniques, and resulting patient outcomes. Relevant case reports may exist that were not included; findings should be interpreted within the context of the selected sample.

**Figure 1:**
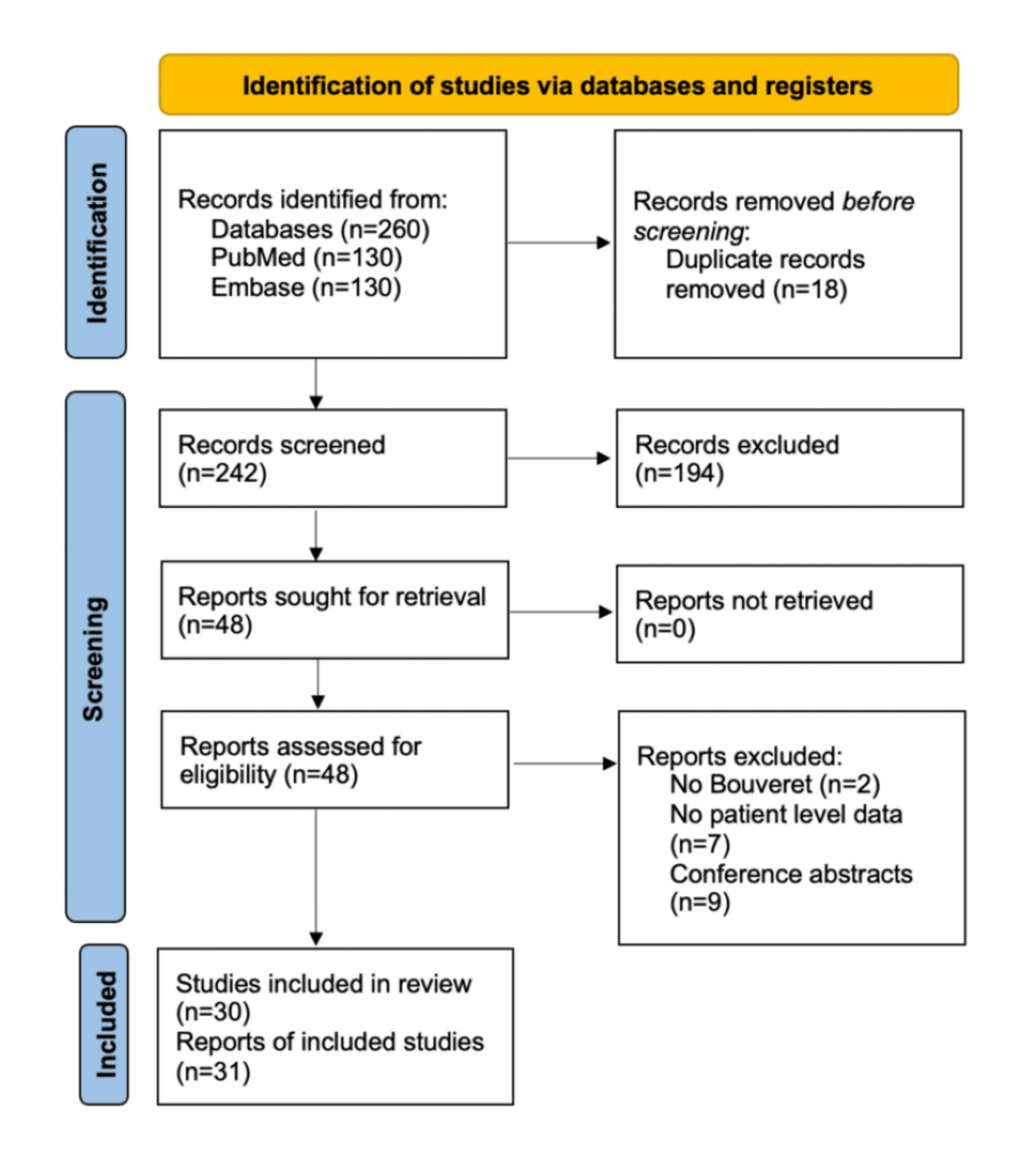
PRISMA Diagram PRISMA: Preferred Reporting Items for Systematic Reviews and Meta-Analyses [13]

Extracted full-text review data were organized into comparative tables and categorized by sex, age, gallstone size, presentation, treatment modality, and outcome. Findings were synthesized to identify common patterns across reports in patient presentation, laboratory values, diagnostic findings, surgeries performed, complications, and treatment effectiveness. The study selection process is summarized in Figure 1.

Data analysis

All included studies of this review consisted of case reports; therefore, analysis was limited to descriptive statistics. Variables (patient age and gallstone size) were reported as means, and categorical variables (procedure used, stone location, patient sex, patient outcome) were summarized as counts and proportions. No regression modeling or meta-analysis was performed. Study quality was assessed using the Joanna Briggs Institute (JBI) critical appraisal checklist for case reports [14]. Included studies matched high-quality standards, demonstrating clear descriptions and reporting.

Risk of bias and methodological limitations

All included studies consisted of case reports and small case series without comparator groups, limiting the ability to assess causality and increasing susceptibility to reporting and publication bias. While study quality was assessed using JBI, the descriptive nature of the available data and variability in reporting across cases remain important limitations. The search was conducted using two databases, which may have resulted in the omission of studies indexed exclusively in other databases. Although a structured Boolean strategy was applied, the rarity of Bouveret syndrome and reliance on case reports can introduce potential selective reporting, publication bias, and heterogeneity in clinical documentation. These factors limit the strength and generalizability of conclusions from the available data.

## Review

Results

A total of 30 articles comprising 31 patients (n=31) with confirmed Bouveret syndrome were found to meet all inclusion criteria. Some cases demonstrated progression from initial proximal obstruction at diagnosis to distal migration of the gallstone prior to removal. These cases were included when the initial presentation was consistent with Bouveret syndrome to assess disease progression as a factor for endoscopic failure. No overlapping patients between case reports were included. Cases were subsequently stratified by initial intervention into minimally invasive and open surgical approaches. Minimally invasive techniques included endoscopic and laparoscopic approaches, while open surgical techniques included laparotomy with primary stone extraction via enterotomy, gastrotomy, or duodenotomy. Additional procedures, such as cholecystectomy and omentoplasty, were performed in select cases based on clinical context and were recorded separately [15-18]. Biliary procedures to repair fistulas included cholecystectomy. The overall cohort demonstrated a mean age of 69.7 years. A total of 18 patients of the cohort were female (58.1% of the population), while males comprised only 13 cases (41.9%). CT imaging was the most consistently utilized imaging modality in 28 cases (90.3%). Diagnostic endoscopy was the next common diagnostic tool, used in 13 cases (41.9%). X-ray and MRI were employed less frequently, appearing in only 6 cases (19.4%) and 3 cases (9.7%), respectively. Additionally, leukocytosis was found to be the most frequent lab finding, reflected in a total of 12 patients (38.7%). Other frequent lab findings noted included hyperbilirubinemia in nine cases (29.0%), transaminitis in 7 cases (22.6%), and azotemia in 4 cases (12.9%) - all of which indicate biliary obstruction and inflammation. A summary of cases with attempted endoscopy can be seen in Table 1, while a summary of labs can be seen in Table 2.

**Table 1:** Summary of Analyzed Case Reports of Patients With Bouveret Syndrome y/o = years old; X = Technique failed; ✓ = Successful technique;﹣Technique not attempted; M: male; F: female * Successful endoscopic procedure, no complications. The patient was referred to a surgeon for laparoscopic disconnection of the biliodigestive fistula and cholecystectomy upon recovery. ** The patient was described as being in her 60s.

Author	Patient Age and Gender	Successful Minimally Invasive Stone Removal	Successful Invasive Surgery for Stone Removal
Chatterjee et al., 2024 [15]	75 y/o, F	x	✓
Datta et al., 2024 [16]	77 y/o, M	✓	x
Philipose et al., 2019 [17]	78 y/o, M	x	✓
Satchithanandha et al., 2023 [18]	79 y/o, F	x	✓
Arian et al., 2024 [19]	54 y/o, M	x	✓
Hufkens et al., 2023 [20]	73 y/o, F	✓	﹣
Chen et al., 2025 [21]	65 y/o, M	x	✓
Ignjatovic et al., 2025 [22]	76 y/o, F	x	✓
Ignjatovic et al., 2025 [22]	72 y/o, F	x	✓
Glover et al., 2025 [23]	79 y/o, M	✓	﹣
Manivannan et al., 2024 [24]	55 y/o, F	x	✓
Khan et al., 2025 [25]	62 y/o, F	x	✓
Kovacheva-Slavova et al., 2024 [26]	49 y/o, M	✓	✓ *
Sabir et al., 2024 [27]	85 y/o, F	﹣	✓
Qian et al., 2024 [28]	64 y/o, M	x	✓
Parisi et al., 2025 [29]	64 y/o, F	✓	﹣
Wang et al., 2019 [30]	61 y/o, F	x	✓
Yang et al., 2025 [31]	60 y/o, M	✓	✓
Probert et al., 2022 [32]	74 y/o, F	x	✓
Chaves et al., 2022 [33]	87 y/o, M	x	✓
Thant et al., 2023 [34]	84 y/o, F	x	✓
Jaroenlapnoppar et al., 2023 [35]	74 y/o, M	✓	﹣
Osorio-Euan et al., 2024 [36]	58 y/o, F	﹣	✓
Patchipalla et al., 2025 [37]	73 y/o, M	﹣	✓
Sousa et al., 2025 [38]	68 y/o, F	﹣	✓
Louis et al., 2025 [39]	31 y/o, F	﹣	✓
Flores-Olmos et al., 2024 [40]	83 y/o, M	﹣	✓
Saeed et al., 2023 [41]	68 y/o, M	﹣	✓
Atri et al., 2024 [42]	82 y/o, F	﹣	✓
Amri et al., 2023 [43]	92 y/o, F	﹣	✓
Smith et al., 2022 [44]	60 y/o, F**	﹣	✓

**Table 2:** Summarized Lab Results

Lab Findings	Number of Cases
Elevated bilirubin	9
Elevated liver enzymes	7
Elevated white blood cell count	12
Elevated urea	4

Therapeutic endoscopic management

Within the minimally invasive category, there were 21 cases meeting the criteria, with the cohort consisting of older patients with a slight female predominance. Stone characteristics were reviewed to identify anatomic or morphological similarities. The duodenum emerged as the most frequent stone location during removal, found in 10 of 21 cases (47.6%). Less frequent stone locations included the ileocecal region and the common bile duct (CBD). Varying regions represent reported sites of stone removal rather than the initial proximal obstruction characteristic of Bouveret syndrome. Minimally invasive cases included 13 cases with documented stone size measurements - median stone size was 4.4 cm (IQR 3.7-5.2 cm; range 2.0-6.6 cm), and mean stone size was 4.37 cm (n = 13). Stone size data were not available for all cases of endoscopic management, as some stones were fragmented or destroyed during removal; therefore, analysis involving stone size was restricted to cases with reported measurements. Cases with missing stone size data were excluded from stone size calculations.

Minimally invasive management demonstrated limited effectiveness, with only 7 of 21 cases (33.3%) achieving initial stone removal using minimally invasive techniques (endoscopy, laparoscopy, and endoscopic retrograde cholangiopancreatography [ERCP]) without the need for immediate additional procedures or conversion to open surgery [16,20,23,26,29,31,35]. A significant 14 out of 21 cases (66.7%) ultimately required conversion to an open surgical approach due to failed endoscopy and laparoscopy removal of the stone. Attempted endoscopy cases can be seen in Table 3, and successful minimally invasive procedures (successful stone removal) can be seen in Table 4.

**Table 3:** Cases With Initial Endoscopic Attempt at Stone Removal Data not reported or not applicable = -; Hx = history; NAFLD: non-alcoholic fatty liver disease; ERCP: endoscopic retrograde cholangiopancreatography; BPH: benign prostatic hyperplasia; HFrEF = heart failure with reduced ejection fraction; M: male; F: female

Author, Year	Sex	Age	Stone Location	Stone size	Endoscopy failure progression	Severity	Comorbidities
Chatterjee et al., 2024 [15]	F	75	Pylorus	-	Endoscopy to open laparotomy with gastrotomy	Gastric outlet obstruction due to a large duodenal gallstone	Elderly age, female, chronic symptoms suggesting delayed diagnosis
Datta et al., 2024 [16]	M	77	Pylorus	-	Endoscopy with electrohydraulic lithotripsy	Coffee ground emesis and abdominal pain	Congestive heart failure and coronary artery disease
Philipose et al., 2019 [17]	M	78	Duodenum	~6.6 cm	Endoscopy with holmium laser and lithotripsy to open surgery	Large obstruction stone	Diabetes, HTN
Satchithanandha et al., 2023 [18]	F	79	Duodenum	~4 cm	Endoscopy to open laparotomy with gastrotomy	Obstruction due to a large gallstone	Elderly age, Female, Hx, cholelithiasis
Arian et al., 2024 [19]	M	54	Ileocecal Junction	4 cm	Endoscopy to open laparotomy with enterotomy, ileal resection and cholecystectomy	Required emergency laparotomy because the patient presented in shock	Presented emergently with shock
Hufkens et al., 2023 [20]	F	73	Duodenum	5 cm	Endoscopic lithotripsy	Duodenal obstruction	Elderly age, Hx gallstones
Chen et al., 2025 [21]	M	65	Duodenum	4 cm	Endoscopy to exploratory laparotomy and cholecystectomy	Progressive obstruction	None reported
Ignjatovic et al., 2025 [22]	F	76	Duodenum	4 cm	Endoscopy to laparotomy with cholecystectomy and omentoplasty	Progressive obstruction	Elderly age, Hx gallstones and cholecystitis
Ignjatovic et al., 2025 [22]	F	72	Gastroduodenum	6 cm	Endoscopy to laparotomy with cholecystectomy and omentoplasty	Progressive obstruction	Elderly age, Hx gallstones and cholecystitis
Glover et al., 2025 [23]	M	79	Duodenum	5.2 cm	Endoscopy with holmium laser and Roth net retrieval	Obstruction due to a large gallstone	None reported
Manivannan et al., 2024 [24]	F	55	-	-	Endoscopic lithotripsy to endoscopic ultrasound-guided gastrotomy and Roux-en-Y surgery	Severe obstruction with bacteremia	None reported
Khan et al., 2025 [25]	F	62	Duodenum	-	Electrohydraulic lithotripsy to laparotomy and gastrotomy	Large gallstone	None reported
Kovacheva-Slavova et al., 2024 [26]	M	49	Common bile duct	-	Endoscopy to endoscopic retrograde cholangiopancreatography and guidewire-assisted papilosphincterotomy, followed by basket extraction. Separate fistula repair surgery, laparoscopy to cholecystectomy	Liver dysfunction due to fistula and choledocholithiasis	None reported
Qian et al., 2024 [28]	M	64	Duodenojejunum	-	Endoscopy to laparoscopic enterotomy	Obstruction	None reported
Parisi et al., 2025 [29]	F	64	Gastroduodenum	3.7 cm	Endoscopy with mechanical lithotripsy	Obstructing stone	None reported
Wang et al., 2019 [30]	F	61	Duodenum	6 cm	Endoscopy to cholecystectomy	Large stone obstruction	None reported
Yang et al., 2025 [31]	M	60	Pylorus		Endoscopic removal of stone; Separate fistula repair surgery, laparoscopy to cholecystectomy	Progressive obstruction, fistula	None reported
Probert et al., 2022 [32]	F	74	Duodenum	2.7 cm	Laparoscopic gastrotomy	Fistula	Parkinson's, multiple sclerosis, asthma, NAFLD, depression, cervical spondylosis
Chaves et al., 2022 [33]	M	87	Pylorus	2 cm	ERCP, laparoscopic gastrotomy	None	Open radical prostatectomy for BPH
Thant et al., 2023 [34]	F	84	Duodenojejunum	5 cm	Endoscopy to enterotomy	None	Hypertension and cholelithiasis
Jaroenlapnopparat et al., 2023 [35]	M	74	Duodenum	3.3 cm	Laser lithotripsy	None	Cardiomyopathy, HFrEF, atrial fibrillation, and ventricular tachycardia
Summary (21 total cases):	Sex:	Average age: 69.6 years	Location:	Average size: 4.37 cm	Percent failure: 66.7%	-	-
-	F: 52.4%	Duodenum: 47.6%	-	-	-	-	-
-	M: 47.6%	Pylorus: 19.0%	-	Percent success: 33.3%	-	-	-
-	-	Duodenojejunum: 9.5%	-	-	-	-	-
-	-	Gastroduodenum: 9.5%	-	-	-	-	-
-	-	Ileocecum: 4.8%	-	-	-	-	-
-	-	Common bile duct: 4.8%	-	-	-	-	-
-	-	Not reported: 4.8%	-	-	-	-	-

**Table 4:** Cases of Successful Minimally Invasive Stone Extraction Data not reported or not applicable = -; Hx = history; HFrEF = heart failure with reduced ejection fraction; M: male; F: female

Author, Year	Sex	Age	Stone Location	Stone size	Endoscopy Course	Severity	Comorbidities
Datta et al., 2024 [16]	M	77	Pylorus	-	Endoscopy with electrohydraulic lithotripsy treatment	Coffee ground emesis	Congestive heart failure and coronary artery disease
Hufkens et al., 2023 [20]	F	73	Duodenum	5 cm	Endoscopic removal of stone; separate fistula repair surgery, laparoscopy to cholecystectomy	Duodenal obstruction	Elderly age, Hx gallstones
Glover et al., 2025 [23]	M	79	Duodenum	5.2 cm	Endoscopy with holmium laser and Roth net retrieval	Obstruction due to a large gallstone	None reported
Kovacheva-Slavova et al., 2024 [26]	M	49	Common bile duct	-	Endoscopy to endoscopic retrograde cholangiopancreatography and guidewire-assisted papilosphincterotomy, followed by basket extraction. Separate fistula repair surgery, laparoscopy to cholecystectomy	Liver dysfunction due to fistula and choledocholithiasis	None reported
Parisi et al., 2025 [29]	F	64	Gastroduodenal junction	3.7 cm	Endoscopy with mechanical lithotripsy	Obstructing stone	None reported
Yang et al., 2025 [31]	M	60	Pylorus	-	Endoscopic removal of stone; separate fistula repair surgery, laparoscopy to cholecystectomy	Progressive obstruction, fistula	None reported
Jaroenlapnopparat et al., 2023 [35]	M	74	Duodenum	3.3 cm	Laser lithotripsy	None	Cardiomyopathy, HFrEF, atrial fibrillation, and ventricular tachycardia
Summary (7 total cases):	Sex:	Average age: 68.0 years	Location:	Average size: 4.3 cm	-	-	-
Female: 28.6%	-	Duodenum: 42.8%	-	-	-	-	-
Male: 71.4%	-	Pylorus: 28.6%	-	-	-	-	-
	-	Common bile duct: 14.3%	-	-	-	-	-
			Gastroduodenal junction: 14.3%	-	-	-	-

Invasive surgical management

Invasive surgical management cohort (n=24) included cases of failed endoscopic management requiring open surgical intervention, as well as cases in which endoscopic management was not attempted due to a high likelihood of failure, with patients proceeding directly to open surgery.

Failed minimally invasive management was defined as the inability to achieve stone removal using endoscopic or laparoscopic techniques, including endoscopic retrograde cholangiopancreatography (ERCP). Cases with successful endoscopic stone removal were excluded from the invasive surgical cohort, regardless of subsequent interventions (e.g., fistula repair).

Among the cases of failed minimally invasive technique requiring conversion to open surgery (n=14), cholecystectomy was the most commonly performed operative intervention, as reported in 6 out of 14 cases (42.9%). Gastrotomy and enterotomy were each conducted in 4 out of 14 cases (28.6%). These cases were further stratified based on the type of open surgical procedure performed. A summary of failed endoscopic management is presented in Tables 5-7.

**Table 5:** Cases with Failed Minimally Invasive Management Requiring Conversion to Gastrotomy Data not reported or not applicable =  -; Hx = history; EHL: electrohydraulic lithotripsy; M: male; F: female

Author, Year	Sex	Age	Stone Location	Stone size	Endoscopy Course	Severity	Comorbidities
Chatterjee et al., 2024 [15]	F	75	Pylorus	-	Endoscopy to open laparotomy with gastrotomy	Gastric outlet obstruction from large duodenal gallstone	Elderly age, female, chronic symptoms
Satchithanandha et al., 2023 [18]	F	79	Duodenum	4 cm	Endoscopy to open laparotomy with gastrotomy	Obstruction from large gallstone	Elderly age, female, Hx cholelithiasis
Manivannan et al., 2024 [24]	F	55	-	-	Endoscopic lithotripsy to endoscopic ultrasound-guided gastrotomy and Roux-en-Y surgery	Severe obstruction with bacteremia	Not reported
Khan et al., 2025 [25]	F	62	Duodenum	-	EHL to laparotomy and gastrotomy	Large gallstone	Not reported
Summary (4 total cases):	Sex:	Average age: 67.8	Location:	-	-	Severity:	-
	Female: 100%	-	Duodenum: 50%	-	-	Large obstruction	-
	M: 0%	-	Pylorus: 25%	-	-	-	-
	-	-	Not reported: 25%	-	-	-	-

**Table 6:** Cases With Failed Minimally Invasive Management Requiring Conversion to Enterotomy Data not reported or not applicable = - ; F: female; M: male

Author, Year	Sex	Age	Stone Location	Stone size	Endoscopy Course	Severity	Comorbidities
Arian et al., 2024 [19]	M	54	Ileocecal Junction	4x4 cm	Endoscopy to open laparotomy with enterotomy, ileal resection and cholecystectomy	Required emergency laparotomy because presented in shock	None reported
Sabir et al., 2024 [27]	F	85	Duodenojejunal junction	3.7 cm	Endoscopy to enterotomy and cholecystectomy	None	None reported
Qian et al., 2024 [28]	M	64	Duodenojejunal junction	-	Endoscopy to laparoscopic enterotomy	None	None reported
Thant et al., 2023 [34]	F	84	Duodenojejunal junction	5 cm	Endoscopy to enterotomy	None	Hypertension and cholelithiasis
Summary (4 total cases):	Sex:	Average age: 71.8 years	Location:	Average size: 4.2 cm	-	-	-
	F: 50%	-	Duodenojejunum: 75%	-	-	-	-
	M: 50%	-	Ileocecal Junction: 25%	-	-	-	-

Among cases that progressed from minimally invasive management to cholecystectomy (n = 6), patients were generally older, with a relatively balanced sex distribution. The duodenum was the most common site of obstruction, while other locations were infrequent. Notably, 4 of 6 cases (66.7%) of this subgroup were found to have had joint procedures (omentoplasty, ileal resection, enterotomy) following cholecystectomy, suggesting complex or multifocal disease. Additionally, 2 of 6 (33.3%) of this subgroup had a prior history of gallstones and cholecystitis [19,21,22,27,30]. A summary of these cases can be seen in Table 7.

**Table 7:** Cases With Failed Minimally Invasive Management Requiring Conversion to Cholecystectomy Data not reported or not applicable =  - ; Hx = history; M: male; F: female

Author, Year	Sex	Age	Stone Location	Stone size	Endoscopy Course	Severity	Comorbidities
Arian et al., 2024 [19]	M	54	Ileocecal junction	4x4 cm	Endoscopy to open laparotomy with enterolithotomy, ileal resection, and cholecystectomy	Required emergency laparotomy because patient presented in shock	None reported
Chen et al., 2025 [21]	M	65	Duodenum	4 cm	Endoscopy to exploratory laparotomy and cholecystectomy	Progressive obstruction	None reported
Ignjatovic et al., 2025 [22]	F	76	Duodenum	4 cm	Endoscopy to laparotomy with cholecystectomy and omentoplasty	Progressive obstruction	Elderly age, Hx gallstones and cholecystitis
Ignjatovic et al., 2025 [22]	F	72	Gastroduodenum	6 cm	Endoscopy to laparotomy with cholecystectomy and omentoplasty	Progressive obstruction	Elderly age, Hx gallstones and cholecystitis
Sabir et al., 2024 [27]	F	85	Duodenojejunal junction	3.7 cm	Endoscopy to enterotomy and cholecystectomy	None reported	None reported
Wang et al., 2019 [30]	F	61	Duodenum	6cm	Endoscopy to cholecystectomy	Large stone obstruction	None reported
Summary (6 total cases):	Sex:	Average age: 68.6 years	Location:	Average size: 4.62 cm	Dual procedure (cholecystectomy + another procedure): 4	Severity:	Comorbidities:
	F: 66.7%	-	Duodenum: 50%	-	Omentoplasty: 2	Progressive obstruction: 66.7%	History of gallstones and cholecystitis: 33.3%
	M: 33.3%	-	Duodenojejunal junction: 16.7%	-	Ileal resection: 1	-	-
	-	-	Ileocecal junction: 16.7%	-	Enterotomy: 1	-	-
	-	-	Gastroduodenum: 16.7%	-		-	-
	-	-		-	-	-	-
	-	-		-	-	-	-

A subset of patients proceeded directly to open surgical management without an attempted minimally invasive approach (n = 10). The duodenum was the most common site of obstruction. The average stone size in this population was 3.8 cm. Notably, 7 of 10 patients (70.0%) underwent preoperative nasogastric (NG) tube decompression, a step not observed in cases managed with minimally invasive techniques. Additionally, hypertension and diabetes were common comorbidities in this group, present in 6 of 10 (60.0%) and 4 of 10 (40.0%) cases, respectively [27,36-44]. A summary of non-endoscopic cases is presented in Table 8.

**Table 8:** Open Surgical Management Without Prior Endoscopic Attempt Data not reported or not applicable = - ; Hx = history; M: male; F: female

Author, Year	Age	Sex	Stone Location	Stone size	Progression	Severity	Comorbidities
Sabir et al., 2024 [27]	85	F	Duodenojejunum	3.7 cm	Enterotomy	None reported	None reported
Osorio-Euan et al., 2024 [36]	58	F	Duodenum	4 cm	Nasogastric decompression to exploratory laparotomy and enterotomy	None reported	Hx hypertension and gallstones
Patchipalla et al., 2025 [37]	73	M	Duodenum	3 cm	Nasogastric decompression to exploratory laparotomy and gastrotomy	Gastric outlet obstruction with bile duct inflammation	Hypertension, diabetes mellitus
Sousa et al., 2025 [38]	68	F	Jejunum	3.8 cm	Nasogastric tube decompression to enterolithotomy with enterorrhaphy	Severe jejunal obstruction; presented in shock	Type 2 diabetes mellitus, hypertension, hyperlipidemia, chronic kidney disease
Louis et al., 2025 [39]	31	F	Duodenum	-	Nasogastric decompression, exploratory laparotomy, duodenal repair	Obstruction with hepatic abscess	Gestational diabetes, hepatic abscess
Flores-Olmos et al., 2024 [40]	83	M	Duodenum	4 cm	Nasogastric decompression, laparotomy, and enterotomy	Duodenal obstruction	Type 2 diabetes mellitus, hypertension
Saeed et al., 2023 [41]	68	M	Duodenum	3 cm	Gastrotomy, cholecystoduodenal fistula repair, cholecystectomy, omentoplasty	Gastric outlet obstruction	Hypertension, hyperlipidemia, cholelithiasis
Atri et al., 2024 [42]	82	F	Duodenum	4 cm	Nasogastric tube, exploratory laparotomy, enterotomy, Gastroenteroanastomosis	Gastric outlet obstruction, dehydrated	Coronary artery disease, gallstones
Amri et al., 2023 [43]	92	F	Common bile duct	4 cm	Exploratory laparotomy, gastrotomy	Fistula	Type 2 diabetes mellitus
Smith et al., 2022 [44]	60	F	Duodenum	5 cm	Nasogastric, exploratory laparotomy, gastrostomy, jejunotomy	None reported	Hypertension, lymphedema, arthritis, gout, suspected left-sided ovarian malignancy
Summary (10 total cases):	Average age: 70.0 years	Sex:	-	Average size: 3.8 cm	Outcomes:	-	-
	-	F: 70.0%	-	-	Nasogastric tube decompression=70%	-	-
	-	M: 30.0%	-	-	No nasogastric tube decompression: 30%	-	-

Discussion

Bouveret syndrome is an uncommon variant of gallstone ileus that is characterized by a more proximal obstruction occurring in the gastric outlet or duodenum secondary to a cholecystoenteric fistula [1,8]. The syndrome is prevalent mainly in elderly females and commonly exhibits symptoms similar to small bowel obstruction and gallstone disease, making diagnosis challenging [6,8]. Current literature frequently recommends initial upper gastrointestinal endoscopic management to be attempted before open surgery due to lower morbidity and mortality rates [11,12]. Multiple published articles described ongoing controversy regarding whether fistula repair should be performed concurrently with stone removal, staged as an individual procedure, or deferred altogether [4,5,11]. However, fistula management was not included as a primary endpoint in this review, and the included studies did not report consistent data to make meaningful comparative analysis on this subject [15-18].

Of the 31 total patients (n=31) included in the cohort, 21 underwent attempted endoscopic stone extraction. Successful removal was achieved in 7 cases (33.3%), corresponding to an endoscopic failure rate of 66.7% (14/21 cases) [15-36]. Female sex comprised 58.1% of the population, while males comprised only 41.9% of the total population (n=31), reinforcing the previously recognized trends of gallstone disease occurring more frequently in the female population. CT imaging was the most consistently utilized modality, reported in 90.3% of cases, and reliably identified ectopic gallstones and features of gastric outlet obstruction. However, while CT provides strong diagnostic value, imaging findings alone may not reliably predict endoscopic success, as factors such as degree of stone impaction and surrounding inflammation or fibrotic changes may not be fully appreciated radiographically [15-35].

This review found that successful and failed minimally invasive cases demonstrated substantial overlap in stone size, suggesting that stone diameter alone does not reliably predict endoscopic success. Included case reports propose that factors such as gallstone composition, local inflammation, anatomical variation, degree of fibrosis, presence of edema, and scar tissue development at the obstruction site may all influence the likelihood of successful endoscopic retrieval [15-35]. Additional patient-level characteristics, including age, past medical history, comorbidities, gastrointestinal tract size, and variation in gastrointestinal folds, have also been suggested as potential contributors to endoscopic removal difficulty. Analysis of successful minimally invasive techniques demonstrated similar stone sizes and obstruction locations compared to both failed minimally invasive cases and those managed with primary open surgery. While larger stones remain challenging to remove endoscopically, the degree of impaction may have a greater influence on the success or failure of endoscopic stone retrieval than the size of the stone or the location of the stone. Imaging may not successfully characterize the mentioned features, which could limit its ability to predict endoscopic success. Procedural outcomes may depend on factors beyond radiographic findings, including clinical presentation. Similarly, laboratory abnormalities commonly reflected biliary obstruction and inflammation; however, these findings did not appear sufficient to predict successful operation or distinguish between degrees of inflammation and obstruction [15-35].

Preoperative nasogastric decompression was reported in 70.0% of patients who underwent primary open surgery, whereas this procedure was not reported in any of the cases where minimally invasive techniques were attempted [36-44]. This finding may reflect differences in the severity of obstruction or perioperative practice patterns; however, this association should be interpreted cautiously, given the small sample size and the observation that endoscopic management has been successful in clinically severe presentations and has also failed in non-emergent cases. These findings suggest that while endoscopic therapy remains a reasonable first-line approach, factors such as patient comorbidities and more advanced stone impaction and inflammation may warrant the need for earlier transition to surgical management, though further study is required.

This review is limited by its analysis relying exclusively on case reports, which introduce heterogeneity in clinical presentation, imaging descriptions, and perioperative decision-making. Variability in documentation across individual studies creates discrepancies and limits standardized comparison. Additionally, Bouveret syndrome is a rare condition, and the small sample size restricts the generalizability of findings. Observed endoscopic failure rate reported in this review should be interpreted cautiously, as publication bias inherent to case-report literature may inflate or disproportionately represent a complex of unsuccessful cases. Future studies would benefit from larger pooled analyses with standardized reporting of gastrointestinal remodeling, stone composition, degree of impaction, and comprehensive documentation of patient presentation and existing comorbidities. Consistency in reporting may help identify subgroups more likely to require primary surgical intervention rather than attempt endoscopic stone retrieval.

## Conclusions

Bouveret syndrome remains a rare and clinically challenging condition with significant diagnostic and therapeutic complexity. Findings from this review suggest that endoscopic success may be influenced by a combination of factors, including stone impaction, local inflammatory changes, and overall disease severity - rather than stone size alone. While minimally invasive techniques remain an appropriate first-line therapy in selected patients, the high failure rate observed in this cohort underscores the potential need for earlier consideration of surgical intervention in certain clinical situations, such as patients with advanced disease, significant comorbidities, or evidence of severe inflammation or impaction. However, observations of this review are based on a small set of case reports and should be interpreted as hypothesis-generating rather than definitive conclusions. Future studies with larger sample sizes and standardized reporting are needed to better define the optimal treatment pathways for different patient subgroups.
